# Calcium Sensitive Fluorescent Dyes Fluo-4 and Fura Red under Pressure: Behaviour of Fluorescence and Buffer Properties under Hydrostatic Pressures up to 200 MPa

**DOI:** 10.1371/journal.pone.0164509

**Published:** 2016-10-20

**Authors:** D. Schneidereit, H. Vass, B. Reischl, R. J. Allen, O. Friedrich

**Affiliations:** 1 Institute of Medical Biotechnology, Friedrich-Alexander-University Erlangen-Nuernberg, 91052 Erlangen, Bavaria, Germany; 2 School of Physics and Astronomy, University of Edinburgh, Edinburgh EH9 3FD, Scotland, United Kingdom; 3 Erlangen Graduate School in Advanced Optical Technologies (SAOT), Friedrich-Alexander-University Erlangen-Nuernberg, 91052 Erlangen, Bavaria, Germany; Russian Academy of Medical Sciences, RUSSIAN FEDERATION

## Abstract

The fluorescent Ca^2+^ sensitive dyes Fura Red (ratiometric) and Fluo-4 (non-ratiometric) are widely utilized for the optical assessment of Ca^2+^ fluctuations *in vitro* as well as *in situ*. The fluorescent behavior of these dyes is strongly depends on temperature, pH, ionic strength and pressure. It is crucial to understand the response of these dyes to pressure when applying calcium imaging technologies in the field of high pressure bioscience. Therefore, we use an optically accessible pressure vessel to pressurize physiological Ca^2+^-buffered solutions at different fixed concentrations of free Ca^2+^ (1 nM to 25.6 μM) and a specified dye concentration (12 μM) to pressures of 200 MPa, and record dye fluorescence intensity. Our results show that Fluo-4 fluorescence intensity is reduced by 31% per 100 MPa, the intensity of Fura Red is reduced by 10% per 100 MPa. The mean reaction volume for the dissociation of calcium from the dye molecules $\Delta_{d}\bar{v}$ is determined to -17.8 ml mol^-1^ for Fluo-4 and -21.3 ml mol^-1^ for Fura Red. Additionally, a model is presented that is used to correct for pressure-dependent changes in pH and binding affinity of Ca^2+^ to EGTA, as well as to determine the influence of these changes on dye fluorescence.

## Introduction

Biochemical reactions in living cells and organisms are strongly influenced by changes in environmental variables such as pH, ionic strength, temperature and pressure. In particular, protein molecules that mediate processes such as cell metabolism and ion homeostasis are sensitive to environmental changes [1]. Although the impact of temperature and pH on chemical equilibria, activation energies and reaction volumes is well-known for many enzymatic biochemical reactions, the effect of pressure as an environmental variable of biochemical equilibria, has only been elucidated in selected enzymatic or cellular systems [2–4]. This is surprising, as environments at high hydrostatic pressures comprise more than 70% of our biosphere. Moreover, high pressure conditions are of great interest for applications in high pressure biosciences and -biotechnology [5, 6].

Several cellular signaling molecules need to be tightly regulated within narrow concentration ranges in living cells. This is of utmost importance, for instance, for Ca^2+^ ions that act as a pleiotropic second messenger to regulate gene transcription, enzymatic reactions, energy metabolism and motor protein interactions among many other processes [7]. To maintain intracellular Ca^2+^ levels in a healthy range, cells contain a huge variety of Ca^2+^-binding proteins, storage organelles and ion channels and pumps. In particular, Ca^2+^ binding proteins act as Ca^2+^-buffers. As such, they redirect Ca^2+^ ions that enter cells or that are being released from intracellular stores. This causes a higher Ca^2+^ saturation at binding sites with a high affinity to Ca^2+^ ions while those with lower binding affinity saturate less at given Ca^2+^ concentrations. The binding of Ca^2+^ ions to Ca^2+^-binding proteins is governed by the law of mass action. Thus, its affinity for association/dissociation to/from the proteins is described by the association/dissociation constant K_a,d_ in the steady-state. This binding/unbinding equilibrium is strongly influenced by environmental parameters, such as pressure.

Most of our knowledge on cellular Ca^2+^-homeostasis stems from live-cell fluorescence imaging. In this technique, Ca^2+^-sensitive fluorochromes are introduced into the cell’s interior, either by trans-membrane diffusion of a permeant acetoxy-methyl ester form (AM) of the dye or by direct micro-injection of the ionized fluorochrome. The dye is excited by a short-wavelength excitation light source, and the emitted fluorescence light is collected by an optical system. For many Ca^2+^-sensitive dyes, tabulated data of their K_a,d_ values and time constants for their binding and unbinding reactions are available [8]. However, the dependence of these parameters on environmental conditions is usually only given for temperature changes, if at all. Thus, whenever fluorescence imaging experiments in living cells or tissues are attempted under high hydrostatic pressure conditions, we are still facing the unknown pressure-dependence of multiple Ca^2+^-binding buffers present within cells, or of Ca^2+^-chelators such as EGTA, that are often used in artificial solutions, in addition to the fluorescent dye. Such chelators are often used to putatively ‘clamp’the free Ca^2+^-levels to a fixed value in order to assess the pressure-dependence of the dye ‘per se’. When such measurements are attempted, the differential effects of pressure on buffers in a recording system are often ignored. However, the correct interpretation of Ca^2+^-dye fluorescence requires detailed knowledge of the effects of pressure on each constituent in a solution within a cell. This includes effects on pH that in turn also affect the Ca^2+^-buffering properties.

Some limited information is available for a ratiometric UV-excited Ca^2+^-dye, fura-2, at pressures up to 40 MPa [9]. In that study, the effects of pressure on the Ca^2+^ chelator EGTA in the solution were also considered in order to provide a defined free Ca^2+^ concentration for fluorescence interpretation. However, since fura-2 is a dye that requires switching of the excitation wavelength, recording speeds are limited by wavelength switch times. Fluo-4 is a fast, high-affinity Ca^2+^-dye that has been widely used in live cell imaging to determine fast changes in Ca^2+^-homeostasis. Since Fluo-4 is a non-ratiometric dye however, its fluorescence intensity strongly depends on the absolute dye concentration in addition to environmental parameters. One way to overcome some of these constraints of Fluo-4, is to use ratiometric dyes with a similar sensitivity range for Ca^2+^ binding (e.g. Fura Red [10]) and compare different emission wavelengths at one excitation wavelength. The advantages and disadvantages of using ratiometric dyes are elaborated by Bruton et al. [11].

Thus, the correct use of Ca^2+^-dyes in the context of live-cell imaging under high hydrostatic pressures requires a detailed analysis of the effects of pressure on pH and any Ca^2+^-buffers present in a solution, in addition to the Ca^2+^-dye. The aim of our present study was to collect data on the pressure dependence of one of the most commonly used biomimetic Ca^2+^-buffers used in live cell research, EGTA, as well as of two selected Ca^2+^-dyes, Fluo-4 and Fura Red. Based on our results, we present a correction procedure that allows raw fluorescence intensities under high hydrostatic pressure conditions up to 200 MPa to be corrected for changes in dye-K_a,d_, pH and EGTA. This allows us to compute molar reaction volumes for the dye-Ca^2+^ interaction. These results should prove to be invaluable for correctly interpreting fluorescence recordings in living cells under pressure in future applications.

## Materials and Methods

### Experimental setup

**Fig 1:**
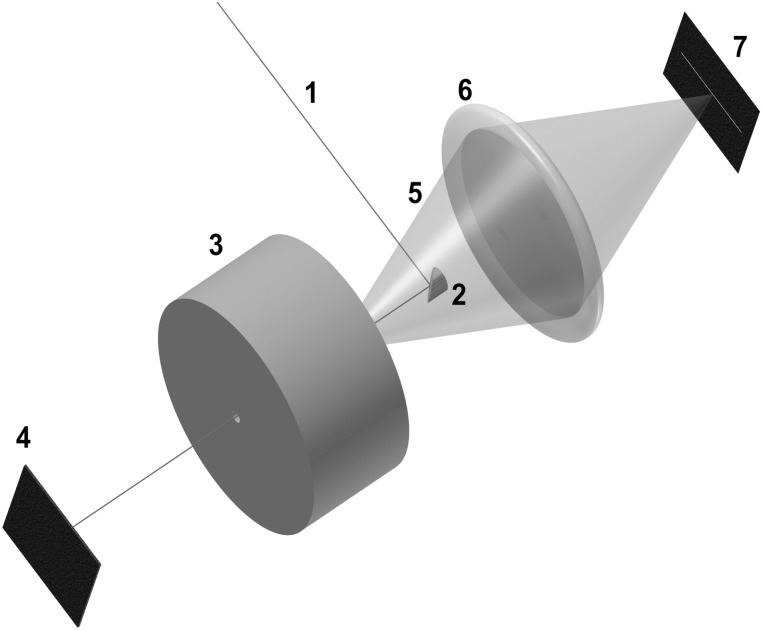
Schematics of the beampath used in the experimental setup. The continuous beam of an Argon laser at a wavelength of 458 nm (1) is deflected by a mirror (2), guided through the high pressure chamber (3) and extinguished in the subsequent beam trap (4). The fluorescent light (5) that is emitted backwards in the direction of the incident laser beam is collected by a condenser lens (6) and focused onto the slit of a three stage spectrometer (7).

The dye sample was sealed into a high pressure chamber containing an optical window as described by Vass et al. [12]. To excite the dye, a continuous Argon laser tuned to a wavelength of 458 nm was used. The laser beam was directed through the pressure chamber by deflecting it from a laser mirror. The fluorescence light, emitted in the backwards direction, was collected by the lens and focused on the slit of a three stage spectrometer. The detector used in the spectrometer was a R943-02 photo-multiplier tube from Hamamatsu. After passing through the high pressure chamber, the excess laser light was extinguished in a beam trap. The experimental setup is illustrated in Fig 1.

**Fig 2:**
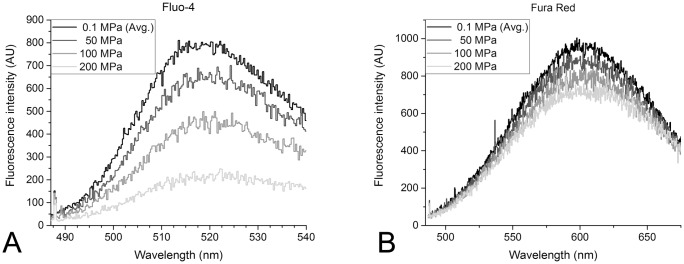
Example of fluorescence emission spectral scans of Fluo-4 (A) and Fura Red (B) at various pressures. The fluorescence intensity *I* is detected by the photomultiplier in arbitrary units (AU). The sharp peak of diamond autofluorescence can be observed at 488 nm in every measurement scan and is used as a wavelength reference. The fluorescence peak of Fluo-4 occurs at 521.5 ±1.5 nm that of Fura Red at 603.4 ±2.4 nm. Scans were taken at 0.1, 50, 100 and 200 MPa of hydrostatic pressure. Scans at 0.1 MPa were taken before and after compression.

Spectroscopic measurements were taken with a spectral resolution of 10 cm^-1^ in wave numbers, which is equivalent to about 0.2 nm in wavelength, starting from 487 nm and ending at 550 nm for Fluo-4 or 700 nm for Fura Red. Diamond, which is used in the windows of the pressure chamber, emits a Raman signal at a spectral distance of −1330 cm^-1^ from the laser wavelength. As this Raman signal occurs within the recording range at about 488 nm, it is used as a reference marker to ensure the correctness of the spectral data. As shown in the example recording in Fig 2, measurements were taken at pressures of 0.1 MPa, 50 MPa, 100 MPa and 200 MPa. The 0.1 MPa recordings were performed prior to compression and after decompression to ensure that no leakage had occurred during pressurisation, and that no bleaching of the dye had been induced by the continuous laser radiation. The chamber was kept at the room temperature of 26 ± 1°C for all measurements.

### Experimental dye solutions

The fluorescent dyes Fluo-4-AM and Fura Red-AM were dissolved in EGTA-buffered physiological solutions containing fixed concentrations of free calcium ions that are commonly used with mammalian muscle cells (i.e. in the sub-*μ*M to the *μ*M range). The final calcium concentration is varied by mixing two physiological stock solutions with high (hCS; ∼ 11 μM) and low (lCS) calcium content at different ratios. The composition of these solutions is given in Table 1. The pH of each solution is adjusted to 7.0 at 23°C by titration with 1 M KOH. The mixing ratios and resulting total calcium concentrations $\left[Ca_{total}^{2+}\right]$ are given in Table 2. The dye concentration in solution is 12 μM for both dyes. The dyes used in this study are AM-ester compounds and thus, have to be activated (de-esterified) prior to our experiments. This is accomplished by adding 10.5 U of pig liver esterase per 500 μl of sample solution. To ensure complete activation of the dyes, the sample solutions are then incubated at room temperature for at least 48 h.

**Table 1:** Composition of physiological stock solutions with low calcium content(lCS) and high calcium content (hCS).

Component	lCS	hCS
	(mM)	(mM)
Hepes	30	30
Mg(OH)_2_	6.3	6.1
EGTA	30	30
CaCO_3_	0	29
Na_2_ATP	8	8
Na_2_CP	10	10

**Table 2:** Calculated free Ca^2+^ ion concentrations at different sample conditions. The values were generated using the software WinMAXC. The WinMAXC simulations were run at the expected pH conditions at respective pressures (pH changes by 0.847 GPa^-1^). The equilibrium constants of the NIST database were also adjusted according to their expected behavior under pressure (logK^EGTA^ changes by -3.6 GPa^-1^).

		Hydrostatic Pressure			
		(MPa)			
		0.1	50	100	200
HCS	$\left[Ca_{total}^{2+}\right]$	Free Calcium			
(%)	(mM)	(μM)			
100	29	11.10	13.60	16.60	24.20
90	26.1	2.81	3.50	4.35	6.73
80	23.2	1.43	1.79	2.23	3.47
75	21.75	1.11	1.38	1.73	2.69
70	20.3	0.88	1.09	1.37	2.13
60	17.4	0.58	0.73	0.91	1.40
50	14.5	0.39	0.49	0.61	0.96
0	0.08	0.00	0.00	0.00	0.00

### Determination of relative fluorescence intensities and dissociation constants

Emission spectra were measured and fitted with the Gaussian Function (1). Only data points that exceed a value of 60% of the maximum intensity recorded in the measurement are considered in the fit. This was done to assure that the asymmetry of the emission peak at lower intensities does not influence the fitting and thereby, change the wavelength parameter of the fitted Gaussian peak. Fitting the curve allows us to extract values for the peak intensity *I*_*peak*_ and peak wavelength *λ*_*peak*_. The parameter for the peak width *ω* is not considered in further calculations.

$$\begin{array}{c} I=I_{peak}\cdot e^{-\frac{{\left(\lambda -\lambda_{peak}\right)}^{2}}{2\cdot w^{2}}} \end{array}$$ (1)

The relative fluorescence intensity $I_{rel}^{p}$ describes the relative change in percent of the peak intensity $I_{peak}^{p}$ at pressure p compared to the peak intensity at atmospheric pressure $I_{peak}^{0.1MPa}$. It is determined according to Eq (2):

$$\begin{array}{c} I_{rel}^{p}=\frac{100\cdot I_{peak}^{p}}{I_{peak}^{0.1MPa}} \end{array}$$ (2)

To eliminate possible shifts in the intensity baseline or detection sensitivity and to facilitate comparison between datasets, normalized fluorescence intensities are used in the determination of the dye dissociation constant *K*_*d*_. The normalized fluorescence intensity $I_{norm}^{p}$ at pressure *p* is determined according to Eq (3), where $I_{peak,min}^{p}$ is the peak intensity for pressure *p* at the calcium concentration with the lowest fluorescence intensity and $I_{peak,max}^{p}$ at the highest, respectively. Note that Fura Red shows its highest peak fluorescence intensity at low calcium concentrations, in contrast to Fluo-4, which shows an increase in fluorescence intensity when the calcium concentration increases.

$$\begin{array}{c} I_{norm}^{p}=\frac{I_{peak}^{p}-I_{peak,min}^{p}}{I_{peak,max}^{p}-I_{peak,min}^{p}} \end{array}$$ (3)

The dissociation constant $K_{d}^{p}$ at pressure *p* is determined by plotting $I_{norm}^{p}$ as a function of the free Ca^2+^ concentration ${\left[Ca_{free}^{2+}\right]}^{p}$ in each sample and fitting the Michaelis-Menten Eq (4) to this data. Eq (4) is obtained from [13]. It is assumed that the dyes are completely saturated with calcium when dissolved in high calcium solution (hCS) which contains a ${\left[Ca_{free}^{2+}\right]}^{p}$ of ∼ 11 μM at 0.1 MPa. Therefore the maximum saturation *v_max_* in Eq (4) can be assumed to have a value of 1.

$$\begin{array}{c} I_{norm}^{p}=(\frac{v_{max}{\left[Ca_{free}^{2+}\right]}^{p}}{K_{d}^{p}+{\left[Ca_{free}^{2+}\right]}^{p}}) \end{array}$$ (4)

The change in relative fluorescence intensity with pressure *ϕ* is defined according to Eq (5) where $\Delta I_{rel}^{p}$ is the change in intensity that is caused by the change in pressure Δ*p*. The overall average change in relative fluorescent intensity $\bar{\phi }$ is determined independently of the total calcium concentration by fitting a linear function to a plot of all $I_{rel}^{p}$ data points as a function of *p*. In this plot, the slope of the fitted function equals $\bar{\phi }$. The calcium dependent average change in relative fluorescent intensity ${\bar{\phi }}_{Ca^{2+}}$ is determined as the slope of a linear function fitted to each set of $I_{rel}^{p}$ data points which share the same total calcium concentration, plotted as a function of *p*.

$$\begin{array}{c} \phi =\frac{\Delta I_{rel}^{p}}{\Delta p} \end{array}$$ (5)

### Dye dissociation constants at high hydrostatic pressure: theoretical considerations

The definition of the dissociation constant $K_{d}^{p}$ for calcium dissociating from a dye at pressure p is given by Eq (6) as the ratio of the free dye concentration [*Dye*_*free*_]^*p*^ and the concentration of the dye-calcium complex [*DyeCa*^2+^]^*p*^ multiplied by the free calcium ion concentration ${\left[Ca_{free}^{2+}\right]}^{p}$.

$$\begin{array}{c} K_{d}^{p}=\frac{{\left[Dye_{free}\right]}^{p}\cdot {\left[Ca_{free}^{2+}\right]}^{p}}{{\left[Dye\;Ca^{2+}\right]}^{p}} \end{array}$$ (6)

$K{}_{d}^{p}$ can be related to the mean molar reaction volume for the dissociation reaction, Δ_*d*_*v*. To derive this relation, we start from the definition of the Gibbs free energy in Eq (7).

$$\begin{array}{c} \Delta G=-S\cdot dT+V\cdot dp \end{array}$$ (7)

Under isothermal conditions, *dT* is zero and the definition of the Gibbs free energy can be simplified as shown in Eq (8).

$$\begin{array}{c} {\left[\frac{\delta G}{\delta p}\right]}_{T}=V \end{array}$$ (8)

For a system in equilibrium, the standard change in molar Gibbs free energy for a reaction $\Delta_{r}G_{m}^{{}^{\circ}}$ is defined according to Eq (9), where T is the absolute temperature in K and R is the gas constant.

$$\begin{array}{c} \Delta_{r}G_{m}^{{}^{\circ}}=-R\cdot T\cdot \ln(K_{d}) \end{array}$$ (9)

Combining Eqs (8) and (9) results in Eq (10) relating the dissociation constant to the molar volume change at dissociation Δ_*d*_*v*.

$$\begin{array}{c} {\left[\frac{\delta (-R\cdot T\cdot \ln(K_{d}^{p}))}{\delta p}\right]}_{T}=\Delta_{d}v \end{array}$$ (10)

Under isothermal equilibrium conditions and discretising the change in pressure, Eq (10) reduces to Eq (11) [14]. The equation is, in principle, similar to the isobaric Arrhenius-equation but describes the pressure-induced change in reaction equilibrium under isothermal conditions. The variable *θ* is introduced as the ratio of the change in the logarithm of the dissociation constant $\Delta \ln(K_{d}^{p})$ and the change in pressure Δ*p*.

$$\begin{array}{c} \theta =\frac{\Delta \ln(K_{d}^{p})}{\Delta p}=-\frac{\Delta_{d}v}{R\cdot T} \end{array}$$ (11)

The molar volume change at dissociation, averaged over multiple measurement sets $\Delta_{d}\bar{v}$, is determined using Eq (12), where $\bar{\theta }$ is the average of *θ* and its value is determined from the slope of a linear fit to the measured values of $\ln(K_{d}^{p})$, plotted as a function of p.

$$\begin{array}{c} \Delta_{d}\bar{v}=-\bar{\theta }\cdot R\cdot T \end{array}$$ (12)

### Estimation of free Ca^2+^ ion concentration at pressure

The binding reaction of a buffer to a ligand is influenced by environmental conditions such as temperature, pH and pressure. As the pH of any solution typically changes with pressure, hydrostatic pressure affects the free calcium ion concentration $\left[Ca_{free}^{2+}\right]$ both directly [15] and through a change in pH [9].

**Fig 3:**
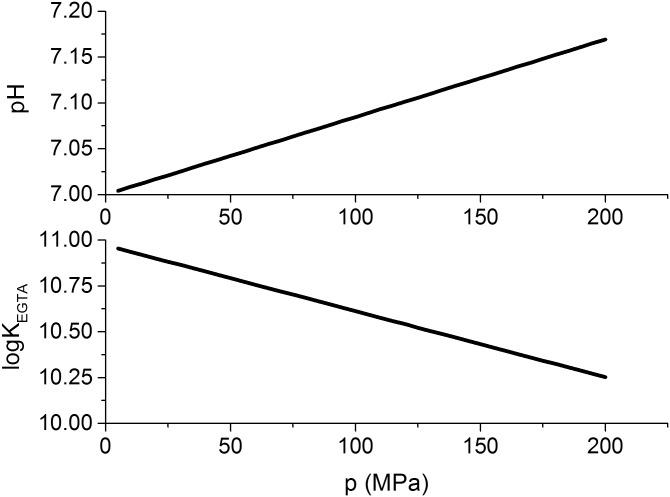
Pressure effects on pH and Ca^2+^-binding to EGTA. Pressure-dependence of pH due to pressure effects on HEPES according to Eq (14) [17] using the Δ_*i*_*v* determined by Kitamura et al. [16]. The development of the equilibrium constant of EGTA binding to Ca^2+^ is estimated by Eq (16) using the Δ_*r*_*v* determined by Hasselbach et al. [15].

The pH buffer HEPES which is used in our solutions has a molar protonic ionization volume of Δ_*i*_*v* = 4.8 ml mol^-1^ [16]. Here, we assume that the change in pH with pressure equals approximately the change in pKa of the buffer [17]. Under this assumption, the change in pH can be estimated to about 0.847 GPa^-1^ by combining the general case of Eq (11) with Eq (13) to obtain Eq (14). The estimated change of pH with pressure, for a HEPES solution at pH 7 at 0.1 MPa is shown in Fig 3(top).

$$\begin{array}{c} \frac{dpH}{dp}≂\frac{dpK_{a}}{dp}=\frac{dlnK_{a}}{dp}\cdot \frac{1}{-\ln10} \end{array}$$ (13)

$$\begin{array}{c} \frac{dpH}{dp}=\frac{\Delta_{i}v}{RT\ln10} \end{array}$$ (14)

The free calcium ion concentration $\left[Ca_{free}^{2+}\right]$ was estimated using the software “WinMAXC v 2.5” by Chris Patton [18]. This program determines the free metal concentration in the presence of chelators by iteratively solving the system of equilibrium reactions within a solution, using binding constants from literature and the absolute concentration of the solution components. In the simulations the pH conditions were adjusted to the estimated pH at the hydrostatic pressure in the sample. The equilibrium constants were taken from the NIST database [19]. The equilibrium reaction of the binding of calcium to the calcium buffer EGTA has a molar reaction volume of Δ_*r*_*v* of 20.4 ml mol^-1^ [15] so that the reaction equilibrium is expected to be shifted towards releasing more free calcium ions under high hydrostatic pressure (i.e. decreasing EGTA binding affinity to Ca^2+^).

**Fig 4:**
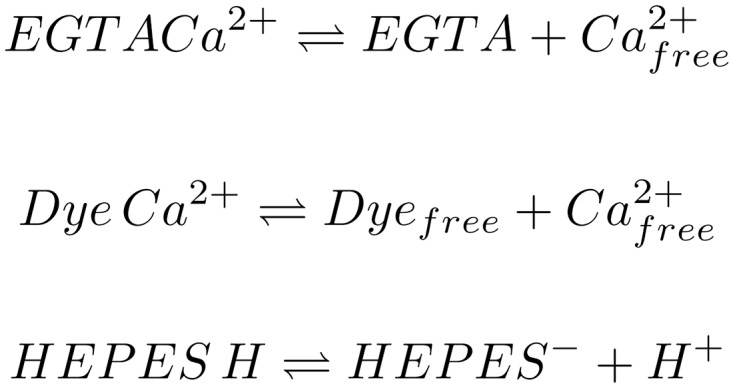
Most important equilibrium reactions in the solution. Shown here are the EGTA-Calcium ion buffer reaction, the Dye-Calcium ion binding reaction and the pH buffer reaction of HEPES. The reaction equilibria are influenced by pressure, temperature, pH and concentration of the components. The whole reaction system influences the observable fluorescence intensity.

**Fig 5:**
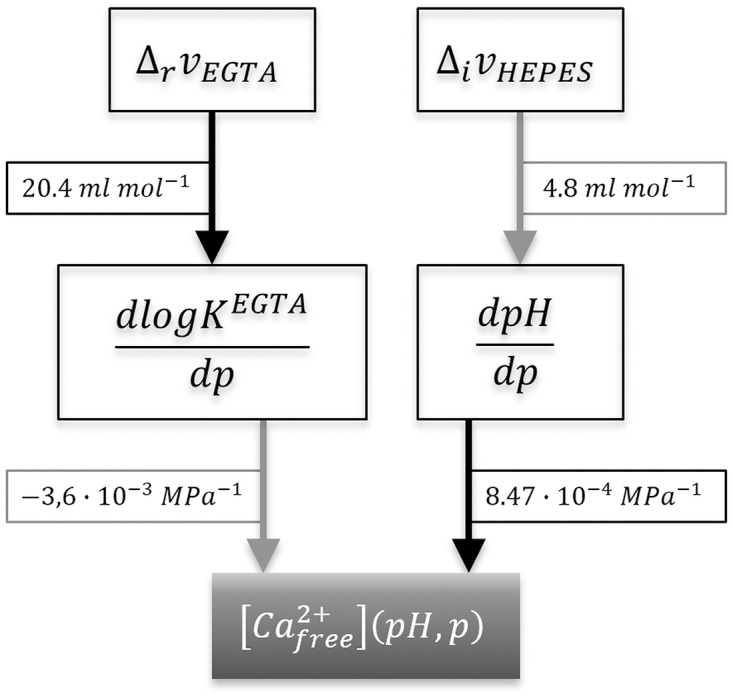
Flowchart illustrating the various effects of rising pressure on $\left[Ca_{free}^{2+}\right]$. A positive influence on the subsequent value is shown as a grey arrow and a negative influence as a black one. The positive reaction volume for the binding of calcium ions to EGTA Δ_*r*_*v*_*EGTA*_ [15] causes the equilibrium constant for the binding reaction *K*^*EGTA*^ to decrease with increasing pressure, which leads to an increase in free calcium ions. The positive protonic ionization volume of HEPES Δ_*i*_*v*_*HEPES*_ causes the pH to rise during a pressure increase, which causes the calcium buffer system to bind more ions and subsequently reduce the free calcium ion concentration with increasing pressure.

The logarithmic equilibrium constant for the binding of Ca^2+^ to EGTA, log *K*^*EGTA*^, is defined according to Eq (15). Its sensitivity to a change in pressure $\frac{\text{dlog}{\text{K}}^{\text{EGTA}}}{dp}$ can be determined as −3.6 ⋅ 10^−3^ MPa^-1^ according to Eq (16). Using this equation, the equilibrium constant for calcium binding that was taken from in the NIST database (log *K*^*EGTA*^ = 10.973), was adjusted to the hydrostatic pressure of the sample. The pressure adjusted values are shown in Fig 3. The effects of pressure on the calcium- and pH buffer system (see Fig 4) and their consequences for the free calcium ion concentration are illustrated in Fig 5, and the estimated free calcium ion concentrations for each pressure are shown in Table 2.

$$\begin{array}{c} \log K^{EGTA}=\frac{\left[EGTA-Ca^{2+}\right]}{\left[EGTA][Ca^{2+}\right]} \end{array}$$ (15)

$$\begin{array}{c} \frac{d\log K^{EGTA}}{dp}=-\frac{\Delta_{r}v_{EGTA}}{RT\ln10} \end{array}$$ (16)

## Results

### Fluorescence intensity profiles at pressure

**Fig 6:**
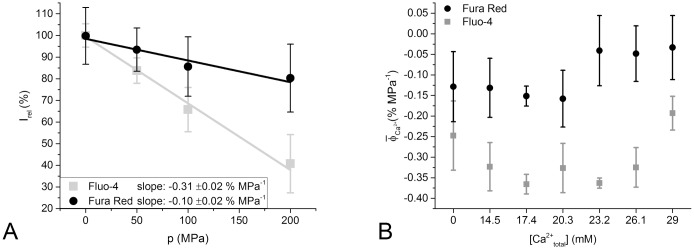
Pressure-dependence of dye fluorescence at different total Ca^2+^ concentrations. A: Mean of relative fluorescence intensity *I*_*rel*_ of all data sets of Fluo-4 and Fura Red, plotted against hydrostatic pressure p. The number of observations *n* for each averaged data point is between 22 and 44, and the standard deviation of the data is displayed as error bars. A linear fit was applied to the averaged data points, the slope of which represents the overall average relative fluorescence change $\bar{\phi }$, determined as −0.308% MPa^-1^ for Fluo-4 and −0.100% MPa^-1^ for Fura Red. B: Mean relative fluorescence change over pressure ${\bar{\phi }}_{Ca^{2+}}$ of the data sets separated by total calcium concentration for Fluo-4 and Fura Red. The number of observations n is 3 to 4 for each box and dye.

We observed no pressure dependence of the wavelength of the Fluo-4 intensity peak (Fig 2). We therefore, focused on analyzing the peak fluorescence intensity changes with pressure. Plotting the peak Fluo-4 intensity as a function of pressure, we did observe a pressure dependent change. Specifically, we found that the relative fluorescence intensity of Fluo-4 changes at a rate $\bar{\phi }$ of -0.308 ±0.017% per MPa of hydrostatic pressure (Fig 6A). This signal change is dependent on the total calcium concentration in the sample solution and thus, the free calcium concentration in the sample solution. The average $\bar{\phi }$ for $\left[Ca_{total}^{2+}\right]$ of 0.0 mM (-0.247% MPa^-1^) and 29 mM (-0.193% MPa^-1^) is significantly different from the average $\bar{\phi }$ at intermediate free calcium ion concentrations (-0.340% MPa^-1^) (P < 0.05, Tukey test; see Fig 6B).

For Fura Red, $\bar{\phi }$ was determined to be −0.100± 0.018% MPa^-1^ as shown in Fig 6A. However, we observed that the pressure-induced signal quenching for Fura Red occurs differently in different ranges of total calcium concentration ($\bar{\phi }$ is significantly different P < 0.05). At total calcium concentrations below 20.3 mM, the signal is reduced at a rate of 0.14% MPa^-1^. However, at total calcium concentrations of 23.2 mM or higher, we observed no significant reduction in peak intensity with pressure. An ANOVA test showed no significant slope of the signal intensity with pressure (P > 0.2). The pressure induced signal quench of Fura Red at total calcium concentrations above 23.2 mM is therefore, considered to be negligible.

### Change in dissociation constant K_*d*_ and determination of mean molar reaction volume $\Delta_{r}\bar{v}$

**Fig 7:**
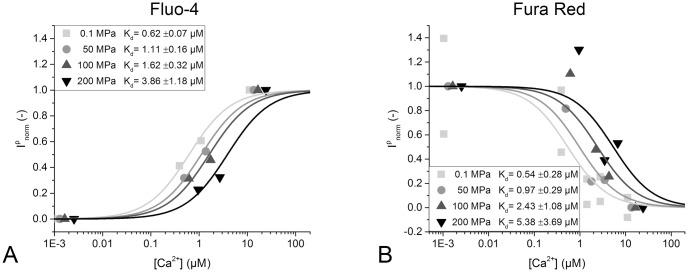
Free Ca^2+^ calibration under high hydrostatic pressures. Graphs of normalized fluorescence intensity $I_{norm}^{p}$ over free calcium ion concentration $\left[Ca_{free}^{2+}\right]$ are fitted with a Michaelis-Menten Eq (4) to determine the dissociation constant of the calcium-dye binding $K_{d}^{p}$ at each hydrostatic pressure *p*. The recordings from one sample batch each are shown for Fluo-4 (A) and Fura Red (B), respectively. The resulting $K_{d}^{p}$ are depicted in each graph and increase with hydrostatic pressure.

To determine the effect of pressure on the dissociation constants of Fluo-4 and Fura Red, the detected fluorescence intensities $I_{peak}^{p}$ for each pressure *p* were normalized such that $I_{peak}^{p}$ is set to zero at a free calcium ion concentration of 1 nM and to unity at 25.6 μM. The normalized fluorescence intensity was plotted as a function of the free calcium ion concentration ($\left[Ca_{free}^{2+}\right]$]), as shown in Fig 7, and fitted with Eq (4). The dissociation constant $K_{d}^{p}$ for each pressure *p* can be directly determined as one of the fitting parameters.

**Fig 8:**
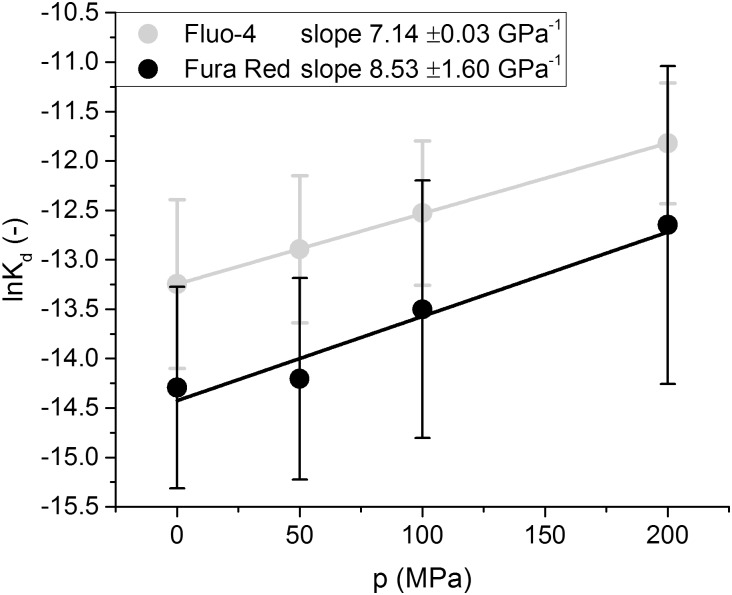
Pressure-dependence of the dissociation constant K_*d*_. The mean ln *K*_*d*_ is plotted against hydrostatic pressure *p* for Fluo-4 and Fura Red, the standard deviation is shown as error bars and the number of observations *n* is 3 for Fura Red and 4 for Fluo-4. The slope *θ* of the linear fit to the data was used to determine the average molar volume change for the dissociation reaction Δ_*d*_*v* by using Eq (12).

Once $K_{d}^{p}$ has been determined, plotting $K_{d}^{p}$ as a function of the hydrostatic pressure *p*, as is done in Fig 8, allows us to determine the mean molar reaction volume as the slope of the plotted values according to Eqs (11) and (12). The calculated $\Delta_{r}\bar{v}$ for Fluo-4 was −17.8 ml mol^-1^ and the calculated $\Delta_{r}\bar{v}$ for Fura Red was −21.3 ml mol^-1^. Those values are comparable to those that have been measured for metal ion chelators and other calcium sensitive fluorescent dyes. (Hasselbach et al.[15] determined the calcium binding reaction volume of Ca^2+^ and EDTA to be 17.8 ml mol^-1^ and Ryan et al.[14] described the dissociation volume of Calcium from Indo-1 to be -23.5 ml mol^-1^)

In an average experiment at 200 MPa, the pressure effects would reduce the fluorescence intensity of Fluo-4 by 60% and increase the logarithmic K_d_ by 1.4 as compared to atmospheric pressure. The ln *K*_*d*_ for Fura Red is increased by 1.7 whereas its fluorescence intensity decreases by 20% under these high pressure conditions.

## Discussion

**Fig 9:**
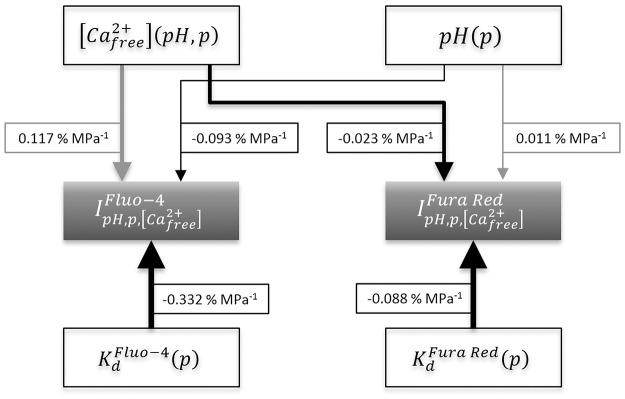
Flowchart of the pressure dependent system variables and their influence on the measured dye fluorescence intensity of Fluo-4 and Fura Red. A positive influence is indicated by a grey- a negative influence by a black arrow. The mean overall fluorescence change $\bar{\phi }$ that is caused by each variable is shown as a label on each arrow. The relative magnitude of the influence of each variable is illustrated in the arrow size.

Advances in optical and mechanical engineering in the past two decades have made it possible to construct high pressure vessels suitable for imaging living cells using commercial microscopes [12, 20–22]. In particular, advances in sealing technologies and optical window glasses mean that imaging at pressures exceeding 100 MPa ranges has become possible for living cells [12]. Fluorescent dyes are invaluable for extracting biological information from such studies e.g. to investigate Ca^2+^-homeostasis in living cells under pressure. We have previously reported the first confocal Ca^2+^-fluorescence experiments in living mammalian muscle cells using Fluo-4 and Fura Red for pressures up to 240 MPa [23]. However, to be able to relate measured fluorescence intensities under pressure to the apparent underlying Ca^2+^ concentrations, one has to bear in mind that Ca^2+^-ion/dye interactions are buffer reactions which are themselves sensitive to pressure, and are also influenced by pressure effects on competing buffer reactions and on environmental factors, in particular pH. There is a common misconception in fluorescence live-cell imaging that ‘*large fluorescence values equal large Ca^2+^-concentrations*’. This statement is misleading since fluorescence can also strongly depend on dye concentration (in non-ratiometric dyes, like Fluo-4, at least). Moreover, changes in the measured relative fluorescence intensity *I*_*rel*_ of fluorescent Ca^2+^-dyes are also affected by various accompanying physico-chemical factors. In particular, pressure changes have multiple effects on Ca^2+^ detection, as illustrated in Fig 9. The relevant factors include: pressure-induced changes in pH, free calcium ion concentration (due to changes in the binding strength of Ca^2+^ ions to the Ca^2+^-chelators present), and changes in the calcium binding affinity of the fluorescent dyes ‘*per se*’. The measured mean relative fluorescence change ${\bar{\phi }}_{measured}$ can be described as a sum of the fluorescence changes that are caused by each of these factors (Eq (17)).

$$\begin{array}{c} {\bar{\phi }}_{measured}={\bar{\phi }}_{K_{d}}+{\bar{\phi }}_{pH}+{\bar{\phi }}_{Ca^{2+}} \end{array}$$ (17)

$$\begin{array}{c} \frac{\Delta I_{rel}^{p,pH}}{\Delta p}=\frac{\Delta I_{rel}^{p,pH}}{\Delta pH}\cdot \frac{\Delta pH}{\Delta p} \end{array}$$ (18)

The rate of change in relative fluorescence as a function of pH $\frac{\Delta I_{rel}^{p,pH}}{\Delta pH}$ has been determined to be rather constant by Rohrbach et al. in the range of pH 7 ±0.2 for both dyes [24]. The change for Fluo-4 seemed to be quite prominent with a change in *I*_*rel*_ of −110% per pH unit, whereas Fura Red displayed much weaker pH dependence with a *I*_*rel*_ change of 13% per pH unit [24]. By using the approximate change in pH at a given hydrostatic pressure that is determined by Eq (14), the change in relative intensity as a function of pressure that is solely caused by the change in pH, ${\bar{\phi }}_{pH}$, can be determined from literature data to be −0.093% MPa^-1^ for Fluo-4 and 0.011% MPa^-1^ for Fura Red.

To estimate the contribution to the change in fluorescence intensity that is caused by the pressure dependent change in the free calcium ion concentration in an EGTA-buffered solution, Eqs (2)–(4) are combined to yield Eq (19). Here ${\left[Ca_{free}^{2+}\right]}_{corr}^{p}$ describes the free calcium value corrected for pressure *p* according to Table 2. Using Eq (19) we find that ${\bar{\phi }}_{Ca^{2+}}^{Fluo-4}=0.117$% MPa^-1^ for Fluo-4 ${\bar{\phi }}_{Ca^{2+}}^{Fura\;Red}=-0.023$% MPa^-1^.

$$\begin{array}{c} {\bar{\phi }}_{Ca^{2+}}=\frac{\Delta I_{rel}^{p,[Ca_{free}^{2+}]}}{\Delta p}=\frac{100}{\Delta p\cdot I_{peak}^{0.1MPa}}\cdot (\frac{I_{peak,max}^{p2}-I_{peak,min}^{p2}}{\frac{K_{d}^{p2}}{{\left[Ca_{free}^{2+}\right]}_{corr}^{p2}}+1}-\frac{I_{peak,max}^{p2}-I_{peak,min}^{p2}}{\frac{K_{d}^{p2}}{{\left[Ca_{free}^{2+}\right]}_{corr}^{0.1MPa}}+1}) \end{array}$$ (19)

Finally, we turn to the contribution to mean signal change that should arise from the pressure effect on the dye dissociation constant ${\bar{\phi }}_{K_{d}}$. This amounts to −0.332% MPa^-1^ for Fluo-4 and −0.088% MPa^-1^ for Fura Red, according to Eq (17). These results show, that the pressure sensitivity of the dissociation constant is the predominant effect influencing the fluorescence intensity change of both dyes over the other two discussed effects.

In our current experiments, the average dissociation constant for Fluo-4 at 0.1 MPa, 1.76 μM was found to be much larger than the value of 345 nM provided by the manufacturer (Thermo Fisher). Our measured value is closer to typical dissociation constants that are observed *in situ* in living cells [25]. One explanation for this discrepancy in our cell-free assay could be the fact that we use AM ester forms of the calcium sensitive dyes and subsequently use pig liver esterase for de-esterification *in vitro*. The esterase enzyme or impurities within the lysate probably exhibit an additional calcium binding property that shifts the actual free calcium ion concentration towards lower values. A similar effect has been observed by Kurebayashi et al. [13] for the enzyme Aldolase in combination with Fura Red. It seems reasonable to suppose that this shift of the free calcium ion concentration is a constant base-line shift, as it is unlikely that either the enzyme itself or any impurities present exhibit significant calcium buffering properties. Thus, this contribution may be considered to be negligible at high hydrostatic pressures. Therefore, the impurities would not affect the change in *K*_*d*_ as a function of pressure in our experiments even though they do affect the absolute *K*_*d*_ values.

The standard deviation of the emission peak wavelength for Fluo-4 was ±3.9 nm over all our measurements. Therefore, the peak wavelength seems to be fairly independent of calcium concentration as well as independent of hydrostatic pressure. This implies that one does not need a spectrometer to optically assess calcium levels by Fluo-4 fluorescence, since the spectrum is not shifted by pressure. As a consequence, Fluo-4 can be reliably used in high pressure microscopy using standard epifluorescence or confocal microscopy settings where fluorescence is collected with optical filters in wavelength bands. Such techniques, have for example, previously been used by us in living muscle cells [23]. Note that in this study, quantitative correction for pressure-induced effects on pH and Ca^2+^-buffers (EGTA) was not performed. The approach presented here will make this feasible in subsequent studies.

**Fig 10:**
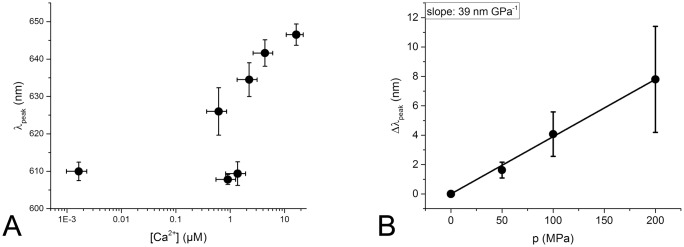
Change in the emission peak wavelength of Fura Red induced by changes in free calcium concentration (A) and pressure (B). A: The wavelength of the emission peak *λ*_*peak*_ of all Fura Red measurements taken at all pressures are grouped by the free calcium concentration [Ca^2+^] of the samples. The emission wavelength does not exhibit a sigmoidal correlation to the free calcium concentration. B: The pressure-induced change in the emission peak wavelength Δ*λ*_*peak*_ of Fura Red compared to the emission peak wavelength at 0.1 MPa of each sample is determined for all samples at all calcium concentrations and plotted over the hydrostatic pressure. The average change in wavelength is with 0.04 nm/MPa negligible within the observed pressure range.

The ratiometric dye Fura Red exhibits a calcium dependent shift in its peak excitation wavelength as well as a smaller calcium dependent shift in its peak emission wavelength. As only one excitation wavelength was available in the experimental setup, it was initially planned to use the calcium dependent shift in emission wavelength for analysis which was expected to be about 25 nm. The acquired experimental data shown in Fig 10A, however, made it clear that the emission wavelength shift does not follow the expected sigmoidal function but rather exhibits a complex correlation towards free calcium concentration that renders the desired evaluation impractical. An analysis of the emission wavelength of Fura Red at different applied pressures (Fig 10B) reveals that a shift is probably existent, but with a magnitude of 0.04 nm MPa^-1^ hardly detectable or relevant compared to a mean emission peak width of around 150 nm. Therefore, the wavelength information in the Fura Red emission spectra was discarded and the dye was evaluated as a non-ratiometric dye with the normalized fluorescence intensity as desired parameter. While this evaluation method provides a rather high variance in data points (see Fig 7B), it still yields values for the dye *K*_*d*_ at 0.1 MPa that are comparable with those of Kurebayashi et al.[13].

For future high pressure analyses, in particular involving cellular systems, our present *in vitro* approach builds a basis which allows exact correction for pressure-induced changes that may strongly affect fluorescence intensities. Uncorrected fluorescence readings may lead to misinterpretation of the effects of pressure on Ca^2+^-homeostasis, or other signaling processes in living cells. Furthermore, it is highly recommended to apply a baro-resistent pH buffer mix, as described by Quinlan et al.[26], to minimize pH effects inside the observed sample. However, as the suggested buffer substances are not among Good’s buffers [27], they may prove to be problematic for live-cell applications. To obtain reliable calcium measurements *in situ*, our results suggest that the use of Fura Red would be a solid choice: either (i) exploit its ratiometric nature and use different excitation wavelengths, as the pressure induced shifts in emission wavelengths appear negligible, or (ii) use it as a non-ratiometric dye and excite only one of its main excitation wavelengths, as the resilience of the dye against pressure induced effects, in terms of fluorescence emission intensity, seems to be high. If Fluo-4 is to be used for *in situ* calcium determination under high pressure, the data in Fig 6A may be useful as a calibration curve to discern between observed effects that are caused by the pressure sensitivity of the dye and those caused by the pressure sensitivity of the sample.

## Supporting Information

S1 Raw Data Archive containing all measured raw data. The provided archive contains all recorded fluorescence intensity raw measurement data. The data is provided as origin project files, separated by sample number and dye type. The naming scheme of the individual spreadsheets is DDCCCSM where DD is the dye type (Fura Red: FR; Fluo-4: F4), CCC is the calcium saturation (00%–100%), S is the sample number and M is the measurement number.
(ZIP)
